# Evaluation of the clinical safety and performance of a narrow diameter (2.9 mm) bone-level implant: a 1-year prospective single-arm multicenter study

**DOI:** 10.1186/s40729-023-00495-x

**Published:** 2023-09-19

**Authors:** Christian Walter, Keyvan Sagheb, Sebastian Blatt, Marcus Oliver Klein, Jan Herrmann, Johannes Kleinheinz, Bilal Al-Nawas

**Affiliations:** 1 Oral and Maxillofacial Surgery of the Mediplus Clinic, Haifa-Allee 20, 55128 Mainz, Germany; 2 Oral and Maxillofacial Surgery, University Medical Center of the Johannes Gutenberg University, Mainz, Germany; 3 Oral and Maxillofacial Surgery Private Dental Office, Stresemannstraße 7-9, 40210 Düsseldorf, Germany; 4 Oral and Maxillofacial Surgery Private Dental Office, Lothar-Streit-Straße 10B, 08056 Zwickau, Germany; 5 Department of Cranio-Maxillofacial Surgery, University Hospital Münster, Albert Schweitzer-Campus 1, 48149 Münster, Germany

**Keywords:** Narrow-diameter implant, Bone-level tapered, Roxolid^®^, Prospective study, Implant survival, PES, Marginal bone-level change, Implant success, Adverse device events

## Abstract

**Purpose:**

Narrow-diameter implants facilitate single‐tooth restoration when interdental or inter-implant spaces and bone volume are inadequate for using standard diameter implants. This study reports the short-term data on the clinical safety and performance of a bone-level-tapered two-piece implant with a 2.9 mm diameter in the clinical practice setting. This study was retrospectively registered on March 1st, 2016 (NCT02699866).

**Methods:**

Implants were placed in partially healed extraction sockets of the central and lateral incisors in the mandible and lateral incisors in the maxilla for single-tooth replacement. The primary outcome was to assess implant survival at 12 months after placement. Secondary outcomes included implant success, pink esthetic score, marginal bone-level changes, and safety.

**Results:**

Twenty four males and 17 females with a mean age of 44.5 (± 18.3 standard deviation) received the implant. Three out of 41 implants were lost yielding a survival rate of 92.7% (95%-CI: 79.0%; 97.6%) at 1 year. One patient reported an ongoing foreign body sensation, pain, and/or dysesthesia at month 12. The average pink esthetic score at 6 months was 11.2 (95%-CI: 10.5; 11.9). The bone level was stable with a mean bone-level change of—0.3 mm (± 0.42 mm standard deviation) at 1 year after implantation. No serious adverse events or adverse device events were reported.

**Conclusions:**

The use of a 2.9 mm diameter bone-level-tapered implant is a safe and reliable treatment option for narrow tooth gaps at the indicated locations. Overall performance and good survival rates support their use in cases, where wider implants are unsuitable.

**Graphical abstract:**

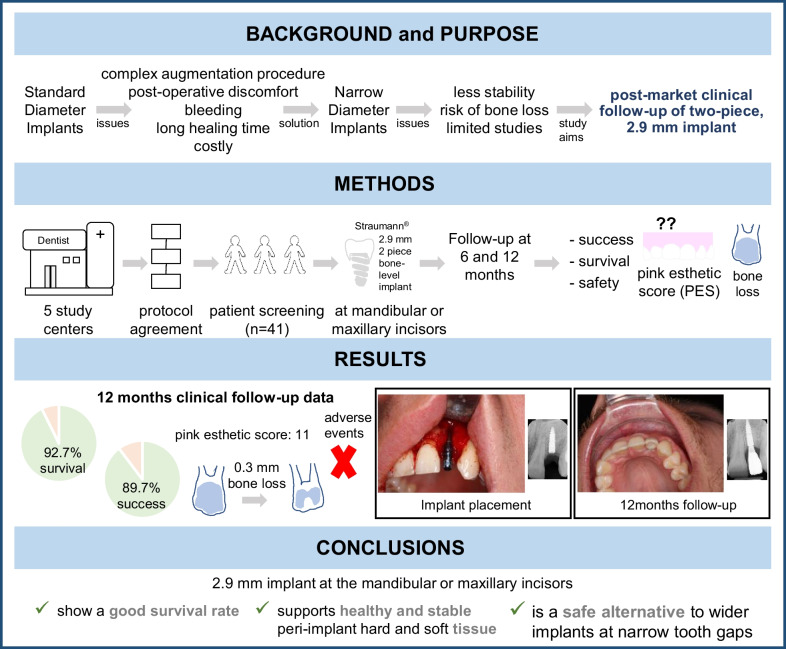

## Background

There are three major treatment options to replace a lost or missing maxillary lateral or mandibular incisor: (a) Orthodontic treatment and substitution with a canine, (b) a resin-bonded fixed dental prosthesis, and (c) an implant [1, 2]. Successful osseointegration and long-term success, function, and esthetics have favored implants over other treatment options in recent years [2, 3]. However, a dental implant requires complex planning and space considerations, such as the evaluation of the width of the edentulous space [2, 3]. For successful implantation of standard diameter implants (SDIs), it is recommended that the horizontal distance between two adjacent implants is supposed to be greater than 3 mm [4]. The distance between an implant and a neighboring tooth should be between 1.5 and 2 mm [5, 6]. In addition, successful implant placement often requires augmentation procedures to ensure sufficient bone volume at the implantation site [7].

Based on the available performance and safety data, narrow-diameter implants (NDIs) can be considered a predictable treatment option with favorable outcomes [8]. In comparison with SDIs, NDIs offer advantages, such as decreased bleeding, reduction in postoperative discomfort, lower costs and improved healing time for patients [9, 10]. They also help to minimize the need [11] or reduce the complexity of lateral bone augmentation procedures (BAP) and may even allow clinicians to conduct simultaneous rather than staged augmentation procedures [12]. Roccuzzo et al. reported that the use of an implant with a diameter of 2.9 mm reduced the frequency of BAP compared to a 3.3 mm implant [13]. Increased prosthetic flexibility may also be possible under certain clinical situations [12]. Furthermore, studies have reported the successful use of 2.7–3.25 mm NDIs and mini-diameter implants as a minimally invasive alternative for patients with insufficient bone ridge thickness in the posterior mandible and a reduced alveolar crest volume [14, 15]. Another meta-analysis study described high patient satisfaction for mini-diameter implants as compared to SDIs when used for implant-supported overdentures [16]. Therefore, in sites with limited bone and narrow spaces, NDIs should be used.

However, NDIs must be used with caution due to some reported limitations. They have lower mechanical stability and fracture resistance [8], resulting in an increased risk of implant or component (abutment or screw) fracture and overload [12]. A study in dogs showed a faster but statistically insignificant progression of induced peri-implantitis with NDIs as compared to SDIs [17]. The decreased diameter reduces the bone-to-implant contact (BIC) surface which might affect the osseointegration of implants [18]. Notably, the fracture fatigue observed in the traditional titanium implants can be avoided by manufacturing new alloys, such as Roxolid^®^, a titanium–zirconium alloy (83–87% titanium (Ti), 13–17% zirconium (Zr) (Institut Straumann AG, Basel, Switzerland) [19]. Chiapasco et al. reported that survival rates of NDIs fabricated with Ti–Zr alloy are comparable to SDIs [19].

The survival rate of NDIs was similar [8, 20], while the mean bone loss was slightly higher than that of SDIs [8, 21]. A systematic review by González-Valls G et al. reported implant survival, success rate, and marginal bone loss at 36 months as 97% (95%-CI: 95.7–98.3%), 96.8% (95%-CI: 94–99.6%), and 0.821 mm, respectively [8]. These values are comparable to other publications reporting on NDIs, such as Klein et al. (survival: 95.6%, success: 93.7%, marginal bone loss: 0.53 mm) [22] and Schiegnitz et al. (survival: 96.5%, success: 96.2%, marginal bone loss 0.993 mm) [7].

According to the group 1 ITI consensus report (2018), implants with diameter ≤ 3.5 mm are generally accepted as NDIs. They are traditionally classified into three main categories as described by Klein et al. in 2014 [22]: (Category 1) implant diameter < 3.0 mm, (Category 2) implant diameter 3.0–3.25 mm, and (Category 3) 3.3–3.5 mm. Implants belonging to categories 2 and 3 are quite often two-piece implants, whereas implants of category 1 are usually one-piece implants, also referred to as “mini-implants (MDIs)”. One-piece implants might need further grinding, so that the implants can be used for dental rehabilitation. This might lead to structural problems in NDIs; however, they support immediate implant placement [22]. Two-piece implants were originally made to facilitate submerged healing and thereby to achieve reduced bone resorption [23]. Furthermore, MDIs are almost exclusively used in edentulous jaws [7]. Some implants classified as category 1 by Klein et al. are two-piece implants with diameters of less than 3 mm in the implant body part but had a greater diameter on the platform level. These implants could also have been included in category 2 [24–26].

In a more recent meta-analysis, Schiegnitz et al. evaluated several studies on NDIs based on the traditional categorization. In addition, due to new product designs of NDIs, such as two‐piece 2.9 mm implants the traditional classification of Klein et al. did not seem adequate anymore. Accordingly, to overcome the observed heterogeneity and bias regarding implant classification the authors proposed a new classification system for NDIs that also considers more precisely the indications [7].

This classification was also considered during the Group 1 ITI Consensus Conference [12]. It categorizes NDIs into Category 1 (diameter of < 2.5 mm (“mini‐implants”), Category 2 (diameter of 2.5 mm to < 3.3 mm), and Category 3 (diameter of 3.3–3.5 mm) [7].

The following indications should be considered for the different categories [12]:

Category 1: Support of definitive complete mandibular overdentures and of interim prostheses, both fixed and removable.

Category 2: Support of definitive complete mandibular overdentures and single tooth replacement in the anterior zone with narrow interdental width (maxillary lateral incisors and single mandibular incisors).

Category 3: Support of definitive complete overdentures and of single tooth replacement in sites with reduced interdental and/or buccal–lingual width.

Importantly, a two-piece 2.9 mm implant will be classified as Category 2 according to Schiegnitz et al., whereas it would belong to Category 1 following Klein´s classification, which should normally contain one-piece implants. As not only the sizes have a relevant influence on performance and safety profiles but also the design [25] one- or two-piece implants should not be mixed in the same categories. However, only limited data for the new implant categories is available.

The present study provides clinical data on the performance and safety of a two-piece narrow-diameter (∅ 2.9 mm) implant in everyday clinical practice at the site of the lateral incisor of the maxilla and the incisors of the mandible and contributes to the new Category 2 of Schiegnitz et al. Implant survival after 12 months was assessed as the primary outcome and implant success, pink esthetic score, marginal bone-level changes, and safety were evaluated as the secondary outcomes. The null hypothesis of the study was that narrow-diameter implants are not a successful and safe treatment option for narrow tooth gaps.

## Methods

This manuscript conforms to the STROBE reporting guidelines.

### Study design

This study was designed as a multicenter, prospective, single-cohort, post-market clinical follow-up (PMCF) study testing a two-piece bone-level-tapered (BLT) implant with a diameter of 2.9 mm (Straumann^®^ Bone-Level-Tapered ∅ 2.9 mm SC, Roxolid^®^, SLActive, Institut Straumann AG, Basel, Switzerland) in the position of the lateral incisor of the maxilla or in any incisor position in the mandible with a follow-up time of 1 year (NCT02699866). The participating sites were all located in Germany and included oral and maxillofacial surgeons in the University Hospital of Mainz (coordinating site), University Hospital of Münster as well as private practices in Mainz, Düsseldorf, and Zwickau. The study protocol was developed in collaboration with all sites and was led by the coordinating site. All participating sites agreed on the final study protocol, including the statistical analysis. The local ethical committees from each state accepted the protocol.

### Study population

The study population consisted of primary male and female patients sampled from those who came to the clinic for regular check-ups (convenience sampling). From October 2015 to December 2019, adults requiring a single tooth replacement with a dental implant in central and lateral incisors in the mandible and lateral incisors in the maxilla were enrolled and included in the study according to the following inclusion criteria: minimum age of at least 18 years, missing tooth in the Federation Dentaire Internationale (FDI) regions 12, 22, 32–42 for at least 4 weeks with natural adjacent teeth or implants (single tooth gap) with a complete soft tissue coverage of the socket.

The following exclusion criteria were applied: inadequate bone volume or quality, local root remnants, inadequate wound healing capacity, incomplete mandibular or maxillary growth, serious internal medical problems, uncontrolled bleeding problems, psychoses, prolonged therapy-resistant functional disorders, xerostomia, weakened immune system, illness requiring periodic steroid use, uncontrolled endocrine disorders, poor general health, drug or alcohol abuse, allergies or hypersensitivity to chemical ingredients of titanium–zirconium alloy, pregnancy or a plan to conceive during the study period. Prior to surgery only one implant per patient was defined as the study implant.

### Study protocol and surgical procedure

**Fig. 1:**
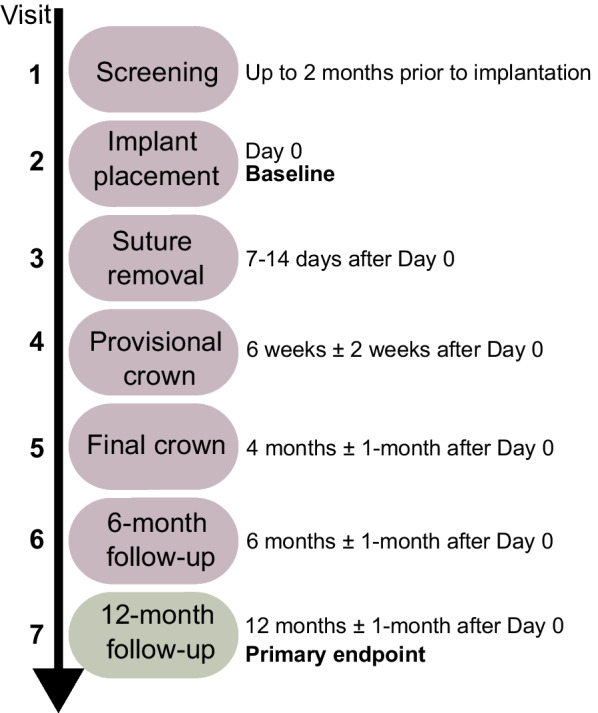
Follow-up visits and treatment protocol. Implants were placed during visit two (day 0 = baseline). A 6-month and 12-month follow-up was conducted adhering to the study protocol

In total seven visits per patient were scheduled during the study. Screening visits were conducted up to 2 months before implant placement. After confirming eligibility, the ∅ 2.9 mm BLT implant was placed according to the manufacturer’s recommendations. Implant lengths used in the study were 10, 12, and 14 mm. Bone augmentation was performed when required. The bone quality (types I–IV), potential bone augmentations (contour, vertical or lateral augmentation), and type of bone augmentation material (autogenous bone graft, xenograft, allograft, synthetic, and others) were recorded. The implant healing procedure was either subgingival (Straumann SC Closure Cap; Institut Straumann AG, Basel, Switzerland) or transgingival (conical Straumann SC Healing Abutment; Institut Straumann AG, Basel, Switzerland). After implant placement, sutures were removed 7–14 days later, and the provisional crown, bonded to a Straumann SC Temporary Crown (Institut Straumann AG, Basel, Switzerland), was placed approximately 6 weeks after surgery. The patients were then referred to a prosthodontist to take the final impression (not a scheduled visit) to finalize the crown. The final crown was bonded to Straumann SC Variobase^®^ abutments (Institute Straumann AG, Basel, Switzerland) and then delivered ~ 4 months after implant surgery and was placed according to each prosthodontist’s routine. A 6-month and a 1-year follow-up were performed, as shown in Fig. 1.

### Data measurement and analysis

In addition to demographic data, dental (reasons for tooth loss, socket preservation history) and medical (current clinically relevant conditions and concomitant medications) history were documented. Smoking history was recorded, and participants were classified as non-smokers, past smokers, and current smokers with up to ten or more than ten cigarettes per day. Time since tooth extraction or loss at the planned study implant site, and type of bone-fill material (autogenous bone, xenograft, allograft, synthetic, and others) were also noted as a part of the pre-operative planning.

The primary analysis was the survival of the implant at month 12. Implant success was defined as the presence of an implant at month 12 and absence of persisting subjective complaints (pain, foreign body sensation, and/or dysesthesia), absence of recurrent peri-implant infection with suppuration, absence of tactile implant mobility, and absence of continuous radiolucency around the implant.

Pink esthetic score (PES) was calculated by assessing seven aspects of the peri-implant soft tissue, such as mesial papilla, distal papilla, soft tissue contours, soft tissue level, alveolar process, soft tissue coloring, and soft tissue texture. If any of the variables of the PES was missing, this variable was assigned a missing value. The soft tissues were evaluated as follows: unnatural–virtually natural–natural, discrepancy: > 2 mm–1–2 mm–< 1 mm, and coloring: clear difference–slight difference–no difference. The alveolar process was evaluated regarding potential resorption and classified as either “clearly resorbed”, “slightly resorbed”, or “no difference”. Each parameter is rated with a 0–1–2 score, where 0 represents the poorest and 2 represents the best score. The highest achievable score is 14.

Radiographs (peri apical X-rays) were taken both at the time of implantation as well as 6 and 12 months after implantation to assess the bone-level changes. The bone level was calculated in millimeters as the mean of the mesial and the distal measurement. Negative bone-level changes represented bone loss between baseline and the respective visit. The relative marginal bone level was assessed as the distance from the implant shoulder to the first bone-to-implant contact (fBIC). For this relative measure of bone change, all subcrestally placed implants had an initial value of fBIC = 0. To include an analysis of initial remodeling, the absolute change from initial marginal bone level to bone level at follow-up was also analyzed. For this absolute measure, subcrestally placed implants had a negative value as measured from implant shoulder to marginal bone level.

Further assessments regarding the course of oral hygiene, plaque index (PI), modified sulcus bleeding index (mSBI), and probing pocket depth (PPD) were undertaken. PI, mSBI, and PPD were assessed on the implant site on the mesial, distal, buccal, and palatal surfaces. PI was scored according to Silness and Loe [27] as follows: Score 0—no plaque, Score 1—a film of plaque adhering to the free gingival margin and adjacent area of the tooth, Score 2—moderate accumulation of soft deposits within the gingival pocket or the tooth and gingival margin, Score 3—abundance of soft matter within the gingival pocket and/or on the tooth and gingival margin. mSBI, was scored according to Mombelli et al. [28] as follows: 0—no bleeding when a periodontal probe is passed along the gingival margin, 1—isolated bleeding spot, 2—confluent red line on margin, and 3—heavy or profuse bleeding.

Data analysis was conducted after all patients completed the 12-month follow-up visit. Two data sets were defined for the analysis: the safety analysis set (SAS) and the full analysis set (FAS). The SAS consists of all patients in the study, who received the study implant. The SAS population was the basis for the safety analysis and provided the baseline characteristics. The FAS consists of all patients in the study, who received the study implant and from whom at least one follow-up measurement after baseline was available. This analysis included patients regardless of any protocol deviations and/or premature termination. Notably, all identified deviations were deemed to have no impact on study integrity, subject’s rights, safety or welfare and none of the deviations were related to an increased risk to the subjects. Hence, no major protocol deviations were identified. The analysis was performed according to the intent-to-treat principle and was applied to primary and secondary endpoints as well as baseline characteristics. To explore the potential impact of bone augmentation on treatment success, all analyses conducted with the FAS or SAS were performed separately for patients with bone augmentation, patients without bone augmentation, and all patients.

### Statistical methods

A descriptive statistical analysis was performed. Implant survival at 1 year was analyzed using the Kaplan–Meier method (including 95% confidence intervals [CI]). Missing values of the implant survival were imputed based on the available data from previous (if implant survival = “no”) and subsequent visits (if implant survival = “yes”). Missing values of other variables were not imputed. Categorical data were analyzed by presenting frequency tables. Implant success, individual success criteria as well as individual PES items were analyzed using frequency tables (including 95%-CI). If missing values were present in frequency analyses, adjusted relative frequencies were calculated. If the fraction of missing values was comparatively large, non-adjusted relative frequencies were reported. For numerical data, the sample statistics mean, standard deviation (SD), median, minimum, and maximum were calculated. PES, bone level, and bone-level changes were analyzed using sample statistics (including 95%-CI for mean). The safety analysis included identifying the number and percentage of patients with at least one adverse event (AE), adverse device effect (ADE), serious adverse event (SAE), serious adverse device effect (SADE), unanticipated serious adverse device effect (USADE), device deficiency (DD) and at least one DD leading to a (S)ADE. Demography, medical history, study procedures, and other baseline characteristics were summarized using descriptive statistics. All analysis was generated using the SAS-software, version 9.2.

## Results

### Demographics

**Fig. 2:**
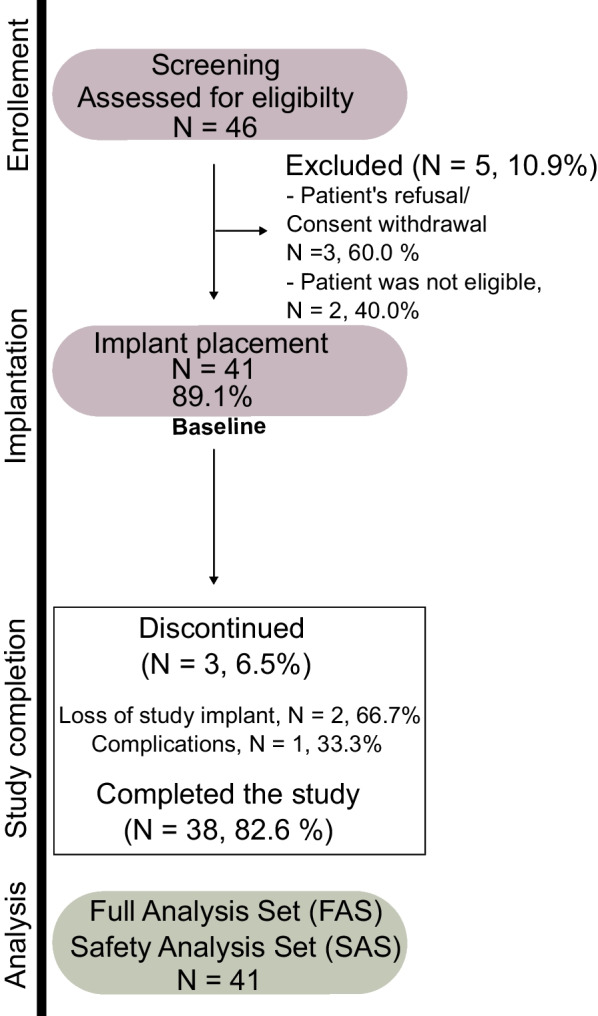
Patient enrollment process and study flowchart. Among 46 patients who were screened for eligibility, 41 patients received the implant and 38 of them completed the study. All 41 patients who received a study implant were valid for the FAS and SAS, since the primary endpoint was analyzed only descriptively and was not affected by the protocol deviations

In total, five study centers included 41 patients (24 male, 17 female) with 41 implants. Please see Fig. 2 for a flowchart showing patient enrollment. The average age of the patients was 44.5 years (± 18.3-year standard deviation [SD]) at the time of implant placement. Hypertonia (*n* = 5), hypothyroidism (*n* = 4), and depression (*n* = 2) were the most common diseases. Thirty-three patients were non-smokers (80.5%), three patients quit smoking at least 17 years ago (7.3%), and five patients were current smokers with less than 10 cigarettes per day (12.2%). The implant sites were toothless for in average 4 months (ranging from 6 weeks to 35 years). Reasons for tooth loss were unsuccessful endodontic treatment (29.3%; *n* = 12), fractured teeth (24.4%; *n* = 10), persistent deciduous teeth with agenesis (12.2%; *n* = 5), trauma (9.8%; *n* = 4), caries (7.3%; *n* = 3), periodontal disease (4.9%; *n* = 2), and unknown reasons in 5 cases (12.2%). Socket preservation was not performed in 30 cases (73.2%). In eight patients (19.5%) different bone fill materials were used such as xenograft [50%, *n* = 4 out of 8], autogenous bone graft [25%; *n* = 2 out of 8], and other [25%; *n* = 2 out of 8]) to preserve the socket at the time of the extraction. Oral hygiene at the baseline was excellent or good in most patients (53.7% and 43.9%, respectively). The mSBI ranged between 0.0 and 1.0, mean mSBI were 0.3 ± 0.5 (mesial) or 0.3 ± 0.5 (buccal, distal and palatal). Table 1 shows a further characterization of patients.

**Table 1:** Demography and baseline characteristics (FAS)

Variable	Bone augmentation (*N* = 14)	No bone augmentation (*N* = 27)	Total (*N* = 41)
Age at baseline (years), mean (SD)	51.44 (15.83)	40.95(18.75)	44.53(18.31)
Gender, *n* (%)			
Male	8 (57.1)	16 (59.3)	24 (58.5)
Female	6 (42.9)	11 (40.7)	17 (41.5)
Number of missing teeth^a^, mean (SD)	7.29 (3.83)	5.93(2.76)	6.39(3.18)
Number of existing implants, *n* (%)			
0	11 (78.6)	27 (100.0)	38 (92.7)
1	1 (7.1)	0 (0.0)	1 (2.4)
2	2 (14.3)	0 (0.0)	2 (4.9)
Number of planned additional implants, *n* (%)			
0	14 (100.0)	18 (66.7)	32 (78.0)
1	0 (0.0)	9 (33.3)	9 (22.0)
Number of clinically relevant conditions (Last 5 years), *n* (%)	14 (100.0)	27 (100.0)	41 (100.0)
Number of current clinically relevant conditions, *n* (%)			
0	9 (64.3)	19 (70.4)	28 (68.3)
1	4 (28.6)	6 (22.2)	10 (24.4)
2	1 (7.1)	2 (7.4)	3 (7.3)
Time since tooth extraction or loss at the planned study site (years), mean (SD)	1.09(2.42)	7.75(11.04)	5.30(9.41)
Number of concomitant medications, *n* (%)			
0	6 (42.9)	14 (51.9)	20 (48.8)
1	2 (14.3)	8 (29.6)	10 (24.4)
2	3 (21.4)	4 (14.8)	7 (17.1)
3	1 (7.1)	1 (3.7)	2 (4.9)
4	2 (14.3)	0 (0.0)	2 (4.9)

*FAS* full analysis set, *SD* standard deviation

^a^The number of missing teeth includes the number of existing implants

### Implant placement-related outcome parameters

**Fig. 3:**
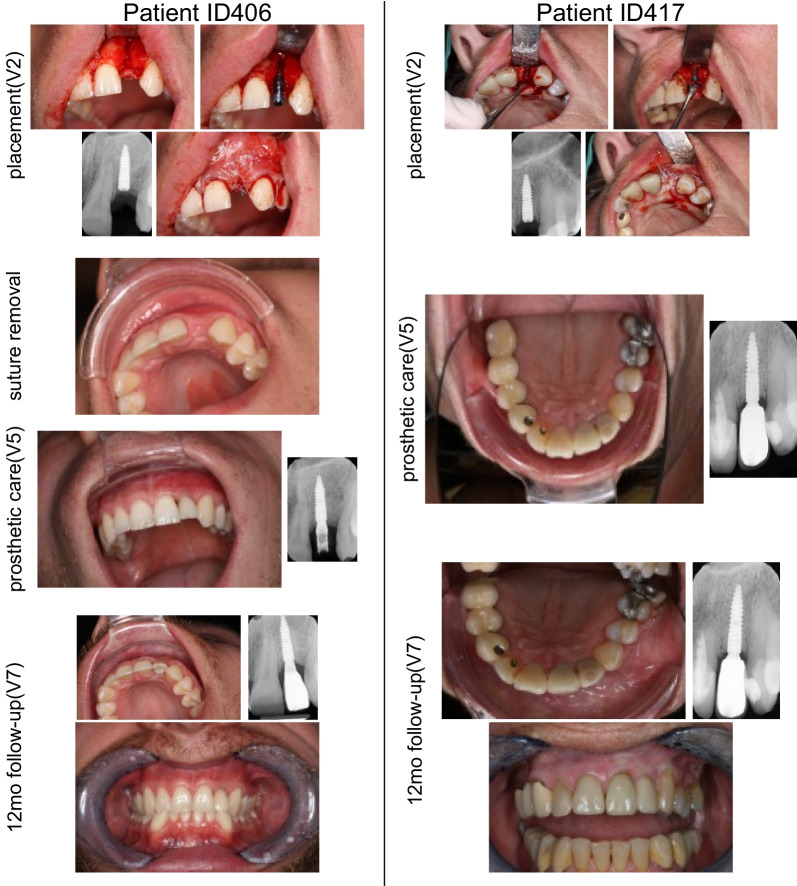
Surgical, follow-up and X-ray images of the representative clinical cases. Clinical and X-ray pictures were taken during implant placement, suture removal, prosthetic care, and 12-month follow-up for 2 representative patients

All surgeries were performed according to instructions and no complications were reported. Study implants were mainly placed at FDI position 12 (18 patients; 43.9%) and 22 (17 patients; 41.5%). 14 cases (34.1%) required a simultaneous bone augmentation procedure. Lateral and contour augmentation was done in 10 (71.4%) and four (28.6%) cases, respectively. In 11 cases (78.6%), a resorbable collagen membrane was used. The inserted implants were either 10 mm (24.4%; *n* = 5), 12 mm (78%; *n* = 32) or 14 mm (9.8%; *n* = 4) in length. The bone quality was assessed as type II (24.4%; *n* = 10), type III (65.9%; *n* = 27), or type IV (9.8%; *n* = 4). Tapping was regarded as not applicable in 75.6% of the patients due to bone quality type III/IV. Primary stability was achieved in 40 out of the 41 patients (97.6%). The further course of the unstable implant was uneventful, and a provisional crown could be installed according to the study protocol. The implants healed submerged in 80.5% (*n* = 33) and transgingival in 19.5% (*n* = 8). The suture was removed in all patients, and 29 patients received a provisional crown and a final crown. The representative surgical and follow-up pictures and X-rays are shown in Fig. 3. The distribution of implant sites and frequency of bone augmentation is presented in Table 2.

**Table 2:** Distribution of implant sites and frequency of bone augmentation

Position of study implant, *n* (%)	Bone augmentation (*N* = 14)	No bone augmentation (*N* = 27)	Total (*N* = 41)
12	5 (35.7)	13 (48.1)	18 (43.9)
22	7 (50.0)	10 (37.0)	17 (41.5)
31	1 (7.1)	0 (0.0)	1 (2.4)
32	0 (0.0)	3(11.1)	3 (7.3)
41	0 (0.0)	1(3.7)	1 (2.4)
42	1 (7.1)	0 (0.0)	1 (2.4)

### Performance analysis of Ø 2.9 mm implant

**Fig. 4:**
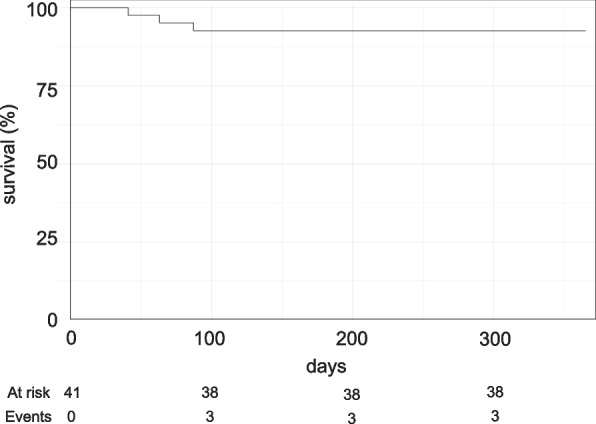
Kaplan–Meier curve. Curve depicting more than 90% survival of the dental implant for up to 1 year of follow-up

**Fig. 5:**
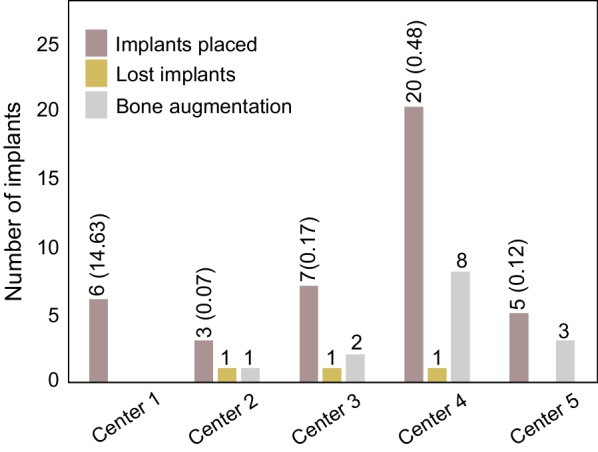
Participating centers and implant loss. The follow-up clinical investigation was performed in five study sites in Germany. The number of implants placed (% values are given above the bar graph), number of lost implants, and number of bone augmentation performed in each study center are shown. Center 1: Department of Oral and Maxillofacial Surgery, Plastic Surgery, University Medical Center of the Johannes Gutenberg-University, Mainz, Center 2: Department of Cranio-Maxillofacial Surgery University Hospital Münster, Center 3: oral surgery group practice at Düsseldorf, Center 4: oral surgery practice at Zwickau, Center 5: dental practice clinic at Haifa-Allee

A total of 41 patients (89.1%) received the implant and 38 of them completed the study (82.6%). The Kaplan–Meier curve of the implants is demonstrated in Fig. 4. Three out of 41 implants were lost before the provisional crown was supposed to be inserted (Table 3; Fig. 5). Therefore, the 1-year implant survival was 92.7% (95%-CI: 79.0%; 97.6%) (Table 4). The implant success rate was evaluated in a total of 39 patients excluding two patients who were lost to follow-up. At 12 months, the implant success rate was 89.7% [95%-CI: 75.8; 97.1]. Four cases reported implant failure which included three cases with implant loss and one case with persisting pain, foreign body sensation, and/or dysesthesia at the end of the 12-month follow-up (Table 5).

**Table 3:** Lost implants

	Implant 1	Implant 2	Implant 3
FDI position	22	22	22
Tooth loss reason	Agenesis	Unsuccessful endo	Fractured tooth
Implant length	10	12	12
Bone quality	III	IV	III
Bone augmentation	No	Contour augmentation No membrane used	no
Primary stable	Yes	Yes	Yes
Healing mode	Subgingival	Subgingival	subgingival

**Table 4:** Implant survival (FAS)

Implant survival	Bone augmentation (*N* = 14)	No bone augmentation (*N* = 27)	Total (*N* = 41)
*N* Total *N* Event *N* Censored	14 1 13	27 2 25	41 3 38
Implant survival (%) [95%-CI]	92.86 [59.08; 98.96]	92.59 [73.50; 98.09]	92.68 [79.00; 97.58]

*CI *confidence interval, *FAS* full analysis set

Implant survival is calculated by the Kaplan–Meier method and censored at the last visit

**Table 5:** Implant success (FAS)

	Bone augmentation (*N* = 14) *n* (%) [95%-CI]	No bone augmentation (*N* = 27) *n* (%) [95%-CI]	Total (*N* = 41) *n* (%) [95%-CI]
6-month follow-up			
No Yes Missing values	1 (8.3) [0.2; 38.5%] 11 (91.7) [61.5; 99.8%] 2	3 (14.3) [3.0; 36.3%] 18 (85.7) [63.7; 97.0%] 6	4 (12.1) [3.4; 28.2%] 29 (87.9) [71.8; 96.6%] 8
12-month follow-up			
No Yes Missing values	1 (7.1) [0.2; 33.9%] 13 (92.9) [66.1; 99.8%] 0	3 (12.0) [2.5; 31.2%] 22 (88.0) [68.8; 97.5%] 2	4 (10.3) [2.9; 24.2%] 35 (89.7) [75.8; 97.1%] 2

*FAS* full analysis set, *CI* confidence interval

The esthetic outcome of soft tissue was evaluated using the PES. The mean PES was 11.6 ± 1.54 with a 95%-CI of [11.1; 12.1] at the 12-month follow-up. Table 6 shows variables of the pink esthetic score and summation scores for follow-up visits at 6 and 12 months.

**Table 6:** Pink esthetic score (FAS)

			6-month follow-up		12-month follow-up	
		Score	*n* (%)	95%-CI	*n* (%)	95%-CI
Mesial papilla	Missing	0	1 (3.3)	0.1; 17.2	0 (0)	0.0; 10.0
	Incomplete	1	21 (70.0)	50.6; 85.3	19 (54.3)	36.6; 71.2
	Complete	2	8 (26.7)	12.3; 45.9	16 (45.7)	28.8; 63.4
Distal papilla	Missing	0	0 (0.0)	0.0; 11.6	0 (0)	0.0; 10.0
	Incomplete	1	20 (66.7)	47.2; 82.7	19 (54.3)	36.6; 71.2
	Complete	2	10 (33.3)	17.3; 52.8	16 (45.7)	28.8; 63.4
Soft tissue contours	Unnatural	0	0 (0.0)	0.0; 11.6	0 (0)	0.0; 10.0
	Virturally natural	1	7 (23.3)	9.9; 42.3	10 (28.6)	14.6; 46.3
	natural	2	23 (76.7)	57.7; 90.1	25 (71.4)	53.7; 85.4
Soft tissue-level discrepancy	> 2 mm	0	1 (3.3)	0.1; 17.2	0 (0)	0.0; 10.0
	1–2 mm	1	5 (16.7)	5.6; 34.7	5 (14.3)	4.8; 30.3
	< 1 mm	2	24 (80.0)	61.4; 92.3	30 (85.7)	69.7; 95.2
Alveolar process	Clearly resorbed	0	0 (0.0)	0.0; 11.6	0 (0)	0.0; 10.0
	Slightly resorbed	1	17 (56.7)	37.4; 74.5	16 (45.7)	28.8; 63.4
	No difference	2	13 (43.3)	25.5; 62.6	19 (54.3)	36.6; 71.2
Soft tissue coloring	Clear difference	0	0 (0.0)	0.0; 11.6	0 (0)	0.0; 10.0
	Slight difference	1	5 (16.7)	5.6; 34.7	9 (25.7)	12.5; 43.3
	No difference	2	25 (83.3)	65.3; 94.4	26 (74.3)	56.7; 87.5
Soft tissue texture	Clear difference	0	0 (0.0)	0.0; 11.6	0 (0)	0.0; 10.0
	Slight difference	1	5 (16.7)	5.6; 34.7	6 (17.1)	6.6; 33.6
	No difference	2	25 (83.3)	65.3; 94.4	29 (82.9)	66.4; 93.4
Pink esthetic score, Mean [95%-CI]			11.2 [10.5; 11.9] SD: 1.83 Median (min; max): 12.00 (6.00; 14.00)		11.6 [11.1; 12.1] SD: 1.54 Median (min; max): 12.00 [7.00; 14.00]	

*FAS* full analysis set, *CI* confidence interval, *SD* standard deviation

**Fig. 6:**
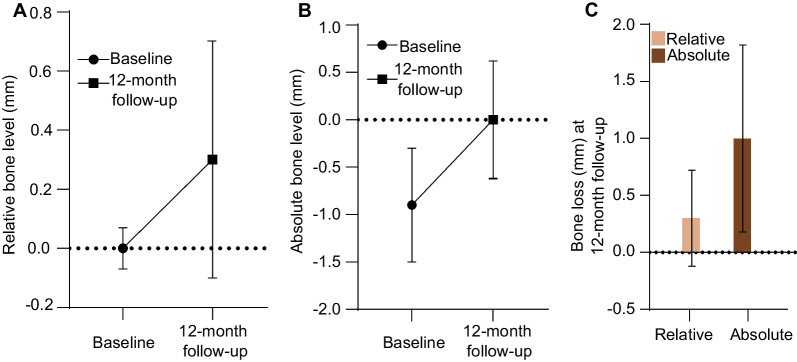
Bone-level changes and bone loss at 12-month follow-up. **A** The relative bone level in millimeters at baseline and 12-month follow-up are given as mean ± SD **B** The absolute bone level in millimeters at baseline and 12-month follow-up are given as mean ± SD **C** Bone loss between baseline and 12-month follow-up are shown. The relative bone-level change between baseline and 12-month follow-up was − 0.3 mm (95%-CI − 0.4; − 0.1) ± 0.42 and the absolute bone-level change was − 1.0 mm (95%-CI − 1.3; − 0.7) ± 0.82. Negative bone-level changes stand for bone loss between baseline and the follow-up

The absolute bone-level changes were obtained for 22 implants after final crown insertion and for 34 implants after a year of implantation. The bone level was reduced by 1.1 mm (SD: ± 0.62 mm, min: − 0.19 mm, max: − 2.54 mm) and 1.0 mm (SD: ± 0.82, min + 0.69 mm, max − 2.86 mm) at the time of final crown insertion (visit 5) and at the 12-month follow-up, respectively. No implant fractures occurred during the 12-month follow-up. The relative bone-level changes resulted in a mean bone-level change of − 0.2 mm (SD: ± 0.29 mm, range: 0.0 to − 0.81 mm) for the timepoint of the final crown insertion and − 0.3 mm (SD: ± 0.42 mm, range: 0.45 to − 1.54 mm) for the 12-month follow-up timepoint. Figure 6 shows the mean bone-level changes of the implants at month 12.

Further assessments included peri-implant soft tissue parameters, such as PI and mSBI. The PI score ranged between 0.1 and 0.4 for the timepoints of the final crown installation and the 12-month follow-up. Scores 2 and 3 were each reached only once at the 12-month follow-up timepoint. The mSBI at the 12-month follow-up ranged from 0.0 on the palatal side to 0.2 on the distal side of the implant.

PPD scores at the 12-month follow-up ranged between 1.0 and 4.0 mm. Median PPD was 2.0 mm at all implant sites and the mean (95%-CI) ± SD values were 1.9 mm (1.7; 2.2) ± 0.71, 1.7 mm (1.5; 1.9) ± 0.7, 1.9 mm (1.7; 2.2) ± 0.8, and 1.7 mm (1.5; 1.9) ± 0.6 on mesial, buccal, distal and palatal implant sites, respectively.

### Safety analysis of Ø 2.9 mm implant

**Fig. 7:**
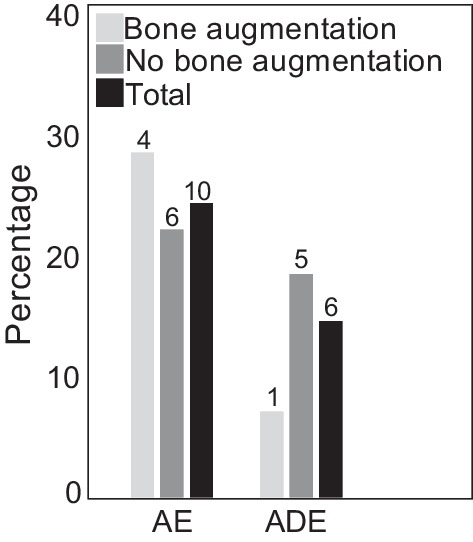
Safety outcomes at month 12. The percentage of patients with adverse events (AEs) and adverse device effects (ADEs) are shown for patients with bone augmentation, patients without bone augmentation, and all patients. Number of patients in each group are shown on top of the bar graph. No SAEs, SADEs, USADEs, and DD were reported

Ten patients (24.4%) experienced a total of 16 AEs, and six patients (14.6%) experienced an ADE. The reported events were not related to the implant in 9 cases, whereas a possible relationship was assessed in 6 cases and a definite relationship in 1 case (foreign body sensation of the implant). Early implant loss after failure to osseointegrate (*n* = 3) and inflammations (*n* = 3) were the most commonly reported complications related to the device (ADEs). Overall, no serious AEs or ADEs or unanticipated SADEs, or device deficiencies were reported. Figure 7 shows the measured safety outcomes post-implantation.

## Discussion

Narrow-diameter implants offer advantages over SDIs in certain treatment scenarios. As mentioned previously, they can be used to avoid bone augmentations [13] or to make implants feasible at all due to the reduced space in the mesio-distal dimension with good long-term results [29]. There seems to be no difference in the crestal bone development between narrow-diameter and SDIs [30]. However, data regarding two-piece NDIs of less than 3 mm diameter is scarce [7], which is addressed in this study.

The observed values of implant survival in this study are within a comparable range reported in the literature. When considering the classification system of 2014 put forth by Klein et al. [22] the implant used in the present study belongs to category 1 with implants smaller than 3.0 mm that are usually one-piece implants. The mean survival rate in this group was 94.7% (range 80–100%) [7]. The most common indication for these very thin implants includes the fixture of a prosthesis in an edentulous mandible. Only three articles described the substitution of an anterior single tooth in this meta-analysis. The survival rates for this scenario were 90.9% [31] and 100% [32] for one-piece implants and 94.2% [33] for a two-piece implant. Furthermore, a retrospective analysis showed that the overall implant survival of MDIs that have diameters ranging from 1.8 mm to 3 mm was 92.1% [34]. Cumulative survival rates of MDIs (diameter ≤ 2.7 mm) and NDIs (diameter: 3–3.3 mm) ranged between 91.17% and 100% in a follow-up of 4 months to 8 years [35]. A recent study in patients receiving a dental implant of 2.9 mm diameter (Straumann BLT implant) reported a survival rate of 100% [36].

In 2018, Schiegnitz et al. [7] proposed a new classification system for NDIs which was also considered during the Group 1 ITI Consensus Conference [12]. It categorizes NDIs into Category 1 (diameter of < 2.5 mm (“mini‐implants”), Category 2 (diameter of 2.5 mm to < 3.3 mm), and Category 3 (diameter of 3.3 mm to 3.5 mm) [7]. Category 2 mostly describes single‐tooth restoration in the anterior region to replace the maxillary lateral or mandibular incisor teeth similar to the implant under investigation. Yet, there are no systematic reviews available for Category 2 (2.5–3.3 mm diameter) implants adhering to the newly proposed classification system and more data within the same indication is required to draw any reliable conclusions. However, the present study on the two-piece 2.9 mm NDI provides clinical data for Category 2 of Schiegnitz et al.

Among the three patients in the present study with implant loss, one was a light smoker with a diagnosis of hypothyroidism. Controlled hypothyroid patients are reported to be at no risk of implant failure [37]. However, whether the patient was medically treated is not known. The second patient used Risperidone to combat depression. Even though users of multiple antidepressant classes are shown to be at a higher risk of implant failure [38], data on second-generation antipsychotic drugs such as Risperidone is limited. In addition, two out of three patients with implant loss had less than 3 months of healing time after extraction, which is shown to negatively affect dental implant survival. An average healing time of 6 months in the maxilla and 3 months in the mandible is recommended [39].

The observed implant success rate of 89.7% [75.8; 97.1%] is consistent with findings of NDIs placed in early (85.8% success) versus delayed (93.3%) protocols [40]. The esthetic outcome of soft tissue parameters including the response and maturation of the soft tissue evaluated by PES was 11.6 ± 1.5 at the 1-year follow-up. There is only one study reporting soft tissue contour changes following single tooth extraction and immediate implant placement. This study reported a mean PES of 12.6 ± 1 at 1 year following implant placement [41] and is comparable to PES reported for equivalent bone-level implants [9.29 (SD: 1.90)] after 2–7-year post-loading [42]. A mean bone-level change of − 0.3 ± 0.4 mm was observed in this study and confirm previous findings investigating how implant diameter affects marginal bone remodeling [43]. Annual vertical bone loss should be less than 1.5 mm in the first year and less than 0.2 mm after 1 year of functional loading to be considered successful [44]. Peri-implant marginal bone-level changes measured for current dental implants range between 0.1 and 0.3 mm at 1 year and are reported with 0.7 and 1.5 mm after 10 years [45] No SAEs or ADEs or unanticipated SADEs, or DDs were reported.

There are several limitations to the present study and the results have to be interpreted cautiously. First, the sample size is small but comparable to the existing literature. The number of patients requiring narrow-diameter implants is fewer, since standard-diameter implants can be used in most cases. The present study tried to address this deficit by increasing the number of participating centers. However, follow-up data were missing for patients from one participating surgical center, since the referring dentist did not provide all the necessary data and the patients did not come back to the surgical center. Second, standardized radiographs were not used in the study. Since the investigation was conducted in a clinical setting, normal panoramic radiographs were taken after surgery in most cases.

Given that studies on two-piece NDIs smaller than 3.0 mm are scarce, the obtained short-term data provides valuable insights. However, further analyses that evaluate the long-term data of implant survival, patient-centered outcomes, and performance comparisons with other implants are still missing. A recent study reported more fenestration defects and a thinner facial bone wall in Straumann BLT implants with a diameter of 3.3 mm compared to 2.9 mm [13]. Still, no statistical difference in terms of marginal crestal bone changes, biological and technical complications, esthetic outcome, or patient-reported outcome measures were identified when using 2.9 or 3.3 mm diameter implants [36]. Findings from the present study show a comparable performance for narrow-diameter implants as known from their wider counterpart (Straumann^®^ BLT) and other titanium implants. This inference needs further validation, since direct comparison to similar devices can only be achieved in a controlled study design. Nevertheless, the data from this prospective, multicenter, observational study in usual clinical practice complement the results achieved in controlled randomized clinical studies and provide critical data on the safety and performance of NDIs. Moreover, results from this study represent the situation in a daily practice setting, unlike a strictly regulated environment, which does not reflect normal treatment situations.

## Conclusions

In conclusion, within the limitations of the present short-term study, the clinical evidence on 2.9 mm narrow-diameter implant (Straumann^®^ Bone-Level-Tapered ∅ 2.9 mm SC, Roxolid^®^, SLActive) supports healthy and stable peri-implant hard and soft tissue with highly aesthetic outcomes. The present study demonstrates that the implant successfully performs its function and is a trustworthy and safe alternative for situations, where wider implants cannot be placed.

## Data Availability

All data and materials are available through Prof. Dr. Dr. Christian Walter.

## Abbreviations

Ø: Diameter
ADE: Adverse device effect
AE: Adverse event
BAP: Bone augmentation procedure
BIC: Bone-to-implant contact
BL: Bone level
BLT: Bone-level tapered
DD: Device deficiency
FAS: Full analysis set
FDI: Federation dentaire international
MBL: Marginal bone loss
MDI: Mini-implant (mini diameter implant)
mSBI: Modified sulcus bleeding index
NDI: Narrow-diameter implant
PES: Pink esthetic score
PI: Plaque index
PPD: Probing pocket depth
SADE: Serious adverse device effect
SAE: Serious adverse event
SAS: Safety analysis set
SDI: Standard diameter implant
USADE: Unanticipated serious adverse device effect
